# Maternal smoking trajectory during pregnancy predicts offspring’s smoking and substance use – The Northern Finland birth cohort 1966 study

**DOI:** 10.1016/j.pmedr.2021.101467

**Published:** 2021-06-23

**Authors:** Ina Rissanen, Markus Paananen, Terttu Harju, Jouko Miettunen, Petteri Oura

**Affiliations:** a Julius Center for Health Sciences and Primary Care, University Medical Center Utrecht and Utrecht University, Utrecht, The Netherlands; b Center for Life Course Health Research, University of Oulu, Oulu, Finland; c Medical Research Center Oulu, Oulu University Hospital and University of Oulu, Oulu, Finland; d Research Unit of Internal Medicine, University of Oulu, Oulu, Finland

**Keywords:** Smoking, Pregnancy, Trajectory, Offspring, Birth cohort, Alcohol

## Abstract

The aim was to characterize the association of maternal smoking trajectory during pregnancy with offspring’s smoking, alcohol and substance use behavior. We used the prospective Northern Finland Birth Cohort 1966 study including 11,653 mothers and their offspring followed up from mothers’ mid-pregnancy to age of 46 years. Main exposure was number of smoked cigarettes per day at each month of pregnancy. Outcome measures were offspring’s smoking, alcohol and drug use at age 14, starting age of smoking, ever-smoking, and smoked pack-years until age 46. Four maternal smoking trajectories during pregnancy were identified with latent class trajectory modelling, namely “non-smokers” (86.0% of mothers), “early quitters” (2.0%), “late quitters” (2.1%), and “consistent smokers” (9.9%). In comparison to non-smokers, all maternal smoking was associated with offspring’s increased odds of lifetime smoking adjusted for sex of the child, father’s smoking, occupational status and place of residence of family, marital status and mood of mother, and desirability of pregnancy. The consistent smoker’s class was associated with offspring’s number of smoked pack years by midlife (median [interquartile range]: 8.3 [1.4–17.4] vs. 4.8 [0.0–13.0], p = 0.028), and alcohol use in young age (odds ratio 1.23 [95% confidence interval 1.05–1.43]). Overall, to prevent parent-offspring transmission of smoking, the cessation support should target women planning pregnancy. Negative effects of maternal continuous smoking during pregnancy include all substance use and reach up to offspring’s middle age.

## Highlights

• Maternal smoking during pregnancy is best represented by a total of four distinct trajectories in this dataset.
• Maternal smoking during pregnancy, even including cessation, predicts offspring’s smoking.
• Maternal smoking trajectory does not predict offspring’s starting age of smoking.
• Mother’s consistent smoking predicts offspring’s smoked pack years by midlife and alcohol use in young age.

## 1 Introduction

Maternal smoking during pregnancy is a well-established risk factor for adverse effects among the offspring (Hartman and Craig, 2018, Clifford et al., 2012, Rogers, 2009, Agrawal et al., 2010). Despite the efforts to reduce smoking during pregnancy, it still remains to be a significant health problem worldwide. The prevalence on pregnancy smoking is around 2% worldwide and 8% in Europe (Lange et al., 2018). Previous studies have found four to seven different trajectories of maternal smoking during pregnancy (Eiden et al., 2013, El-Khoury et al., 2017, Dukes et al., 2017, De Genna et al., 2016). These trajectories represent different pathways and timings of smoking changes during pregnancy.

Parental smoking affects offspring’s smoking habits (Otten et al., 2007, Mays et al., 2014, Chassin et al., 2008, Melchior et al., 2010). Previous studies have shown that especially smoking during pregnancy associates with increased smoking and nicotine dependence among the offspring (Buka et al., 2003). Of previously identified maternal pregnancy smoking trajectories, those representing increasing smoking during pregnancy or smoking cessation only after the childbirth have been associated with increased smoking among the offspring (De Genna et al., 2016). Maternal smoking during pregnancy affects also offspring’s other substance use (Nomura et al., 2011, Porath and Fried, 2005).

Smoking damages general health, causes cancer and chronic obstructive pulmonary disease, exacerbates chronic diseases, and consequently remains one of the leading causes of mortality worldwide (Reitsma et al., 2017, World Health Organization, 2013, World Health Organization, 2009, National Center for Chronic Disease Prevention and Health Promotion (US) Office on Smoking and Health, 2014). Effective tobacco control policies, preventive initiatives and cessation support should build on a comprehensive analysis of factors underlying the smoking habits (National Center for Chronic Disease Prevention and Health Promotion (US) Office on Smoking and Health, 2014, World Health Organization. WHO Framework Convention on Tobacco Control, 2003). Smoking is also associated with increased use of alcohol and other harmful substances (Brook et al., 2012, Nelson et al., 2015, Cance et al., 2017, Lee et al., 2018). Therefore, it is crucial to prevent parent-offspring transmission of smoking at earliest in the life-course.

In this study, we used a geographically representative, unique population-based birth cohort. With a dataset accumulated from the prenatal period until the age of 46, we identified diverging maternal smoking trajectories during pregnancy, and studied their association with offspring’s smoking-related behavior at several time points along the follow-up. Specifically, the aims of this study were to investigate i) changes in maternal smoking during the pregnancy with an advanced trajectory modelling technique, and ii) their association with offspring’s smoking and other substance use behavior until mid-life. We hypothesized that timing of changes in maternal smoking during pregnancy would be reflected in the offspring’s smoking and substance use behavior in later life.

## 2 Material and methods

The Northern Finland Birth Cohort 1966 (NFBC1966) is an unselected population-based birth cohort containing data on 12,055 mothers and their 12,058 babies born alive in the Finnish provinces of Oulu and Lapland with an expected date of birth in 1966 (University of Oulu, 1966). Data collection of NFBC1966 started in the year 1965 when the mothers were pregnant.

Permission to gather data was obtained from the Ministry of Social Affairs and Health, and the study was approved by the Ethical Committee of Northern Ostrobothnia Hospital District in Oulu, Finland. Data protection was scrutinized by the Privacy Protection Agency of Finland. Informed consent was inquired from all the participants according to the prevailing legislations, and those offspring who did not give permission to use their data (n = 59), were excluded from the study. Offspring of multiple deliveries (n = 346) were excluded from the sample. The sample for the current study included 11,653 mothers and their offspring who were born and alive and followed up from mothers’ mid-pregnancy. Multiple imputation procedure was used due to loss of follow-up until the age of 46 years. In total, 10,599 (91.0%) participated at age 14 (participation defined as answering the substance use questions), and 8,520 (73.1%) participated at age 31 and 6,996 (60.0%) participated at age 46 (participation at 31 or 46 years defined as giving informed consent).

### 2.1 Mothers’ smoking

Information on the mothers’ health, sociodemographic factors, and lifestyle habits including smoking was collected by the local midwives in the antenatal clinics using a structured questionnaire (https://www.oulu.fi/nfbc/pregnancyantenatal66) between 24th and 28th gestational weeks. The missing questionnaire data on maternal smoking beyond 28th gestational week were complemented during the pregnancy and after the delivery from maternity clinic cards. Smoking during pregnancy was assessed with questions asking if the mother had smoked at least one cigarette or pipeful per day before the pregnancy and did she change her smoking habits during the pregnancy. If the mother had changed her smoking habits, she was asked if she stopped, increased or decreased smoking and at which pregnancy month the change took place. The mother was also asked how much she smoked daily before and after the change classified to categories 0) no smoking, 1) 1–5 cigarettes, 2) 6–10 cigarettes, 3) 11–15 cigarettes, 4) 16–20 cigarettes, 5) 21 or more cigarettes. The amount of smoked cigarettes (classified from 0 to 5 as listed above) of each pregnancy month was calculated based on information on 1) amount before the change, 2) timing of the change, and 3) amount after the change. This monthly smoking information was used in the trajectory model.

### 2.2 Outcomes

The outcomes of this study were smoking, alcohol use, being drunk, and other substance use at age 14, smoking any time during the follow up (i.e. ever-smoking), starting age of smoking, and smoked pack years until the end of the follow-up.

At the age of 14, NFBC1966 members were asked to report their current smoking status, alcohol use, being drunk, and other substance use in a questionnaire. Smoking was asked with the response options as follows: ‘1) never tried, 2) tried once, 3) tried twice or more, 4) yes, occasionally, 5) yes, approximately twice a week, 6) yes, 1–5 cigarettes a day, 7) yes, 6–10 cigarettes a day, and 8) yes, over 10 cigarettes a day.’ Individuals who chose response options 4–8 at the age of 14 were considered smokers and those who chose options 1–3 were considered non-smokers. Alcohol use was classified as ‘not using’ if they reported not having tried or having tasted only once and as ‘using’ if they reported having drunk alcohol a few times, drinking monthly, or drinking weekly. In addition to that, participants were asked if they have been drunk and were classified as ‘no’ if they answered ‘never’ and as ‘yes’ if they reported having been drunk once or more. Participants were categorized to use other substances (drugs or “thinner”) if they reported having tried them once or more or using them regularly.

In the 31- and 46-year follow-up questionnaires, all participants were asked whether they had ever smoked tobacco during their lives. Tobacco was defined as filter cigarettes, other cigarettes, pipes and cigars. The individuals who reported smoking were asked to report the age at which they had started smoking and the time they have smoked during their lives so far to the accuracy of one year. The 31 years follow-up assessment was selected as the primary source for data on smoking starting age and the 46 years follow-up assessment was used as the primary source for data on smoking ending age to reduce recall bias. Participants were also asked to report the amount of tobacco products they smoke now or used to smoke previously per day. Smoked pack years were calculated based on the information on years smoked and the amount.

### 2.3 Covariates

Covariate variables were obtained from the questionnaires and delivery reports. The covariate variables included sex of the child, father’s smoking, occupational status and place of residence of family, marital status and mood of mother, and desirability of pregnancy. Covariates were selected based on previous literature and primary hypothesis on possible causal pathways.

Sex of the child was obtained from delivery reports. Father’s smoking was asked in the 14 years questionnaire and categorized as current, former, and never smoker; this postal questionnaire was sent first to the children, and if they did not respond, to their parents. Other covariates were collected from pregnancy questionnaires. Socioeconomic status of family was defined as the highest occupational status of the mother or father during the pregnancy, and categorized as unemployed, unskilled worker, skilled worker, and professional. Place of residence was divided in two categories according to population density in which ‘urban’ was defined as any living in a town or village and ‘rural’ as living in the countryside. Mother’s marital status was classified as ‘married’ if reported so, or ‘not married’ if the answer was unmarried, widowed, or divorced. Self-reported mood was asked in the questionnaire as “has the mother’s mood during this pregnancy been i) ordinary, ii) depressed, iii) very depressed”, and was classified to be depressed if the answer was ‘depressed’ or ‘very depressed’. Desirability of pregnancy was classified as ‘unintended’ if mothers reported in the questionnaire to have wished for the pregnancy to come later or not at all.

### 2.4 Statistical analyses

To address missing values of maternal smoking, covariates, and offspring’s substance use outcomes we used multiple imputation using 10 datasets. Overall rate of missing data before multiple imputation was 11.0%. Information on maternal smoking was missing for 4.2% of participants, age of father was missing for 5.8% of participants, other covariates collected during pregnancy were missing for 0.0–2.3% of participants, and father’s smoking status was missing for 15.4% of participants. Offspring’s substance use outcomes collected at age 14 were missing for 9.1% of participants. Outcomes collected later in life, i.e., ever smoking in life, starting age of smoking, and smoked packyears by age 31 or 46 had missing values for 35.4 and 63.4% of participants, respectively. Data were assumed to be missing at random. Sensitivity analyses were conducted on a complete case basis using non-imputed outcome measures. In sensitivity analyses, mother’s pregnancy smoking trajectory classification was based on the imputed data.

Developmental trajectories of maternal smoking during pregnancy were identified using the PROC TRAJ latent class growth modelling (LCGM) macro in SAS version 9.4 (SAS Institute Inc., Cary, NC, USA) (Jones et al., 2001, Andruff et al., 2009, Nagin and Odgers, 2010). The LCGM analysis was based on monthly smoking status (months 1–9) of the pregnant mother. As the smoking data were considered ordinal (0–5 according to intensity of smoking at each month), we used the zero-inflated Poisson-based model (ZIP) in PROC TRAJ. We tested for models with one to six trajectory classes and selected the most suitable model on the basis of several measures of model adequacy (Jones et al., 2001, Andruff et al., 2009, Nagin and Odgers, 2010): 1) Bayesian and Akaike Information Criterion (BIC and AIC, respectively), in which low absolute values imply good fit of data; 2) Bayes Factor (B_10_) and the log form of the Bayes Factor (2log_e_(B_10_) ≈ 2(ΔBIC)), where ΔBIC is the BIC of the alternative (i.e. more complex) model less the BIC of the null (i.e. less complex) model; values > 6 indicate strong evidence against the null model; 3) Posterior membership probability, in which class averages of > 0.70 indicate acceptable fit; and 4) Absolute and relative class sizes, also considering the subsequent analyses. The subjects were classified according to highest posterior membership probability in the most suitable model.

Basic characteristics of maternal smoking trajectories are reported as means or percentages where applicable and compared using analysis of variance (ANOVA) and Chi square test for continuous and dichotomous variables, respectively. Pooled results are presented, and P-values are shown as means of 10 imputed data sets. Logistic regression analysis was used to study the associations between maternal smoking trajectories and dichotomous outcomes: offspring’s smoking status at age 14, offspring’s use of alcohol at age 14, offspring having been drunk at age 14, offspring’s use of other drugs at age 14, and offspring smoking ever in their lives. Generalized linear model was used to study the associations between maternal smoking trajectories and continuous outcomes: offspring’s starting age of smoking, and offspring’s smoked packyears by age 46. The analyses were adjusted for sex of the child, father’s smoking, occupational status and place of residence of family, marital status and mood of mother, and desirability of pregnancy.

IBM SPSS Statistics, version 25.0, for Windows (IBM Corp., Armonk, NY, USA) was used for multiple imputation and statistical analyses except for the trajectory modelling.

## 3 Results

### 3.1 Mother’s smoking trajectories

**Fig 1:**
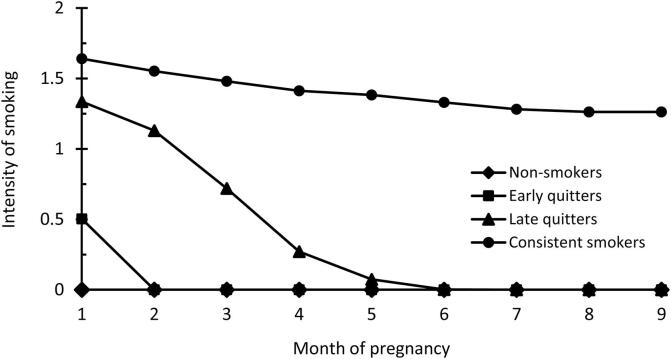
Trajectories for maternal smoking during pregnancy by time: non-smokers (86% of mothers), early quitters (2%), late quitters (2%), consistent smokers (10%). Intensity of smoking is presented as group mean according to classes 0) no smoking, 1) 1–5 cigarettes, and 2) 6–10 cigarettes per day.

Fit parameters of trajectory models with 1–6 classes are presented in Table 1. The four-class model provided the most appropriate interpretation of the data showing better fit than models with 1–3 classes and having sufficient number of participants in each class. Models with 5 and 6 classes converged but were frail, i.e., with at least one unacceptably small or completely empty class. In the four-class model, the first smoking trajectory class were ‘non-smokers’ (n = 10,025, 86.0%) and were used as the reference group in the analyses. Class 2 included ‘early quitters’ (n = 235, 2.0%), class 3 included ‘late quitters’ (n = 245, 2.1%), and class 4 included ‘consistent smokers’ (n = 1148, 9.9%) (Fig. 1).

**Table 1:** Model fit parameters of trajectory models with 1–6 classes.

Number of classes	AIC	BIC	Null model	2log_e_(B_10_)	Class sizes (%)	Average posterior probabilities
1	−514 929.2	−514 948.6	–	–	100	1.00
2	−225 476.4	−225 520.1	1	578 857.0	88.4/11.6	1.00/1.00
3	−216 751.2	−216 819.0	2	17 402.2	2.5/87.5/10.0	0.95/1.00/1.00
4	−216 478.8	−216 570.9	3	496.2	85.9/2.0/2.1/10.0	0.97/0.92/0.95/1.00
5	−215 710.4	−215 826.7	4	1488.4	0.2/85.9/2.0/2.1/9.8	0.98/0.97/0.92/0.95/1.00
6	−215 833.4	−215 974.0	5	−294.6	1.1/-/1.1/87.5/9.7/0.6	0.88/-/0.69/0.65/1.00/0.80

Mothers who smoked during pregnancy (trajectory classes 2–4) were more likely to be younger, unmarried, feel depressed, not wanting the pregnancy, and live in urban areas than non-smoking mothers (Table 2). The fathers of the offspring were also more likely to be younger and to smoke if the mother smoked during pregnancy.

**Table 2:** Basic characteristics of the maternal smoking trajectory classes and the full sample.

	**Non-smokers (n = 10025)**	**Early quitters (n = 235)**	**Late quitters (n = 245)**	**Consistent smokers (n = 1148)**	**Total (n = 11653)**	**P**
	**N (%)/mean (SD)**	**N (%)/mean (SD)**	**N (%)/mean (SD)**	**N (%)/mean (SD)**	**N (%)/mean (SD)**	
**Sex of the child**						0.247
Male	5094 (50.8%)	126 (53.6%)	125 (51.1%)	615 (53.6%)	5961 (51.2%)	
Female	4931 (49.2%)	109 (46.4%)	120 (48.9%)	533 (46.4%)	5692 (48.8%)	
**Father smoking**						< 0.001
Never	2704 (27.0%)	29 (12.2%)	37 (15.3%)	135 (11.8%)	2906 (24.9%)	
Former	3348 (33.4%)	76 (32.1%)	83 (33.8%)	346 (30.2%)	3853 (33.1%)	
Current	3972 (39.6%)	131 (55.7%)	125 (50.9%)	667 (58.1%)	4895 (42.0%)	
**Marital status**						< 0.001
Not married	330 (3.3%)	18 (7.5%)	25 (10.2%)	127 (11.1%)	500 (4.3%)	
Married	9695 (96.7%)	218 (92.5%)	220 (89.8%)	1021 (88.9%)	11,153 (95.7%)	
**Desirability of pregnancy**						< 0.001
Unintended	3629 (36.2%)	87 (36.9%)	89 (36.2%)	525 (45.8%)	4330 (37.2%)	
Intended	6396 (68.8%)	148 (63.1%)	156 (63.8%)	623 (54.2%)	7323 (62.8%)	
**Mood of mother**						< 0.001
Depressed	1365 (13.6%)	43 (18.1%)	38 (15.6%)	226 (19.6%)	1671 (14.3%)	
Normal	8660 (86.4%)	193 (81.9%)	207 (84.4%)	922 (80.4%)	9982 (85.7%)	
**Family SES**						< 0.001
No occupation	74 (0.7%)	3 (1.1%)	3 (1.3%)	24 (2.1%)	103 (0.9%)	
Unskilled worker	3820 (38.1%)	61 (26.1%)	56 (22.7%)	388 (33.8%)	4325 (37.1%)	
Skilled worker	3308 (33.0%)	98 (41.8%)	130 (53.1%)	486 (42.3%)	4022 (34.5%)	
Professional	2824 (28.2%)	73 (31.0%)	56 (22.9%)	251 (21.8%)	3203 (27.5%)	
**Residence**						< 0.001
Urban	3114 (31.1%)	135 (57.3%)	102 (41.5%)	529 (46.1%)	3880 (33.3%)	
Rural	6911 (68.9%)	101 (42.7%)	143 (58.5%)	619 (53.9%)	7773 (66.7%)	
**Age of mother**	28.2 (SD 6.7)	24.9 (SD 5.7)	23.7 (SD 5.7)	25.7 (SD 6.6)	27.7 (SD 6.7)	< 0.001
**Age of father**	31.1 (SD 7.3)	27.6 (SD 6.4)	26.8 (SD 6.4)	28.6 (SD 7.5)	30.7 (SD 7.3)	< 0.001

### 3.2 Mothers’ smoking trajectories and offspring’s substance use

**Fig. 2:**
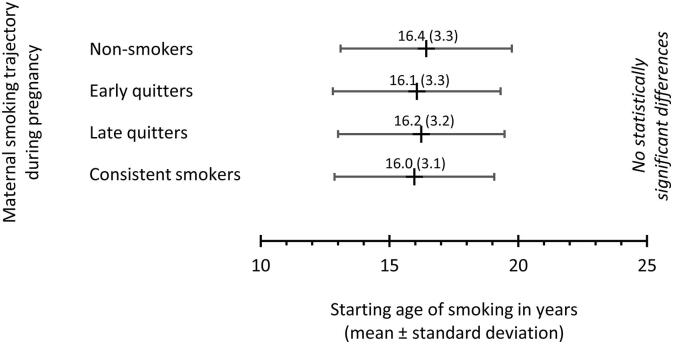
Offspring’s starting age of smoking among maternal smoking trajectory classes.

**Fig. 3:**
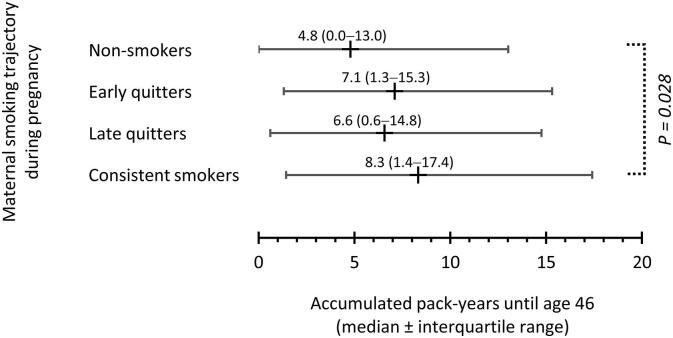
Offspring’s smoked pack-years until age 46 among maternal smoking trajectory classes. The statistically significant between-class difference according to fully adjusted GLM model for natural logarithm -normalized pack years is indicated.

Offspring’s starting age of smoking was similar in all mother’s trajectory classes (Fig. 2). Offspring of smoking mothers (trajectory classes 2–4) had increased odds to ever smoke during their lives up to age of 46 years compared to offspring of non-smoking mothers even when adjusted for sex of the child, father’s smoking, occupational status and place of residence of family, marital status and mood of mother, and desirability of pregnancy (Table 3). Offspring of consistent smokers had smoked more pack years by age 46 (median 8.3 years, interquartile range (IQR) 1.4–17.4) than offspring of non-smokers (median 4.8 years, IQR 0.0–13.0) (p = 0.028) (Fig. 3).

**Table 3:** Maternal smoking trajectories and offspring’s substance use until the age of 14 years and ever smoking until the age of 46 years.

	Smoking at 14 years		Alcohol at 14 years		Being drunk at 14 years		Other drugs at 14 years		Ever smoking	
	N (%)	OR (95% CI)	N (%)	OR (95% CI)	N (%)	OR (95% CI)	N (%)	OR (95% CI)	N (%)	OR (95% CI)
**All (n = 11653)**	2038 (17.5%)	n.a.	2865 (24.6%)	n.a.	3005 (25.8%)	n.a.	122 (1.0%)	n.a.	8663 (74.3%)	n.a.
**Non-smokers (n = 10025)**	1693 (16.9%)	ref	2392 (23.9%)	ref	2499 (24.9%)	ref	89 (0.9%)	ref	7337 (73.2%)	ref
**Early quitters (n = 235)**	57 (24.1%)	1.39 (0.97–1.98)	74 (31.3%)	1.33 (0.91–1.94)	70 (29.9%)	1.14 (0.78–1.65)	6 (2.7%)	2.25 (0.93–5.44)	196 (77.5%)	**1.54 (1.06**–**2.22)**
**Late quitters (n = 245)**	41 (16.6%)	0.90 (0.61–1.34)	60 (24.4%)	0.97 (0.69–1.35)	70 (28.6%)	1.11 (0.82–1.49)	6 (2.6%)	2.14 (0.70–6.56)	200 (81.6%)	1.42 (0.96–2.10)
**Consistent smokers (n = 1148)**	248 (21.6%)	1.16 (0.98–1.36)	340 (29.6%)	**1.23 (1.05**–**1.43)**	365 (31.8%)	**1.23 (1.05**–**1.44)**	20 (1.8%)	1.49 (0.76–2.91)	931 (81.1%)	**1.29 (1.09**–**1.54)**

Compared to non-smoking mothers, offspring of consistent smoker mothers had increased odds to try alcohol (OR 1.23; CI 1.05–1.43) and to be drunk (OR 1.23; CI 1.05–1.44) at age 14 when adjusted for sex of the child, father’s smoking, occupational status and place of residence of family, marital status and mood of mother, and desirability of pregnancy (Table 3).

### 3.3 Sensitivity analyses

In complete case analyses, offspring of early quitters had increased odds to smoke at age 14 (OR 1.57; CI 1.09–2.25), to try alcohol at age 14 (OR 1.43; CI 1.02–1.99), to try other drugs at age 14 (OR 2.86; CI 1.11–7.33), and to ever smoke up to age of 46 years (OR 1.61; CI 1.04–2.49), compared to offspring of non-smoking mothers. Offspring of consistent smoker mothers had increased odds to try alcohol at age 14 (OR 1.26; 1.07–1.49), to be drunk at age 14 (OR 1.34; CI 1.14–1.58), and to ever smoke up to age of 46 years (OR 1.36; CI 1.08–1.68). These sensitivity analyses were adjusted for sex of the child, father’s smoking, occupational status and place of residence of family, marital status and mood of mother, and desirability of pregnancy. Offspring of consistent smoker mothers had lower mean starting age of smoking than offspring of non-smokers (15.9 vs. 16.3; p = 0.034). Mean of logarithmic transformed packyears was lower in offspring of early quitters compared to offspring of late quitters (p = 0.042) and to offspring of consistent smokers (p = 0.045).

## 4 Discussion

In this large and prospective birth cohort study with 46 years follow up, we identified four maternal smoking trajectories that predicted offspring’s smoking and substance use behavior. All maternal smoking during pregnancy – even including cessation – increased offspring’s odds of lifetime smoking. The consistent smoker’s trajectory associated with offspring’s number of smoked pack years by midlife, and alcohol use in young age. Offspring’s starting age of smoking was similar in all maternal trajectory classes. The findings were independent from sex of the child, father’s smoking, occupational status and place of residence of family, marital status and mood of mother, and desirability of pregnancy.

Our interpretation of the results may be limited by a few factors. The main limitation is that the smoking habits of mother after the pregnancy were not known, and social aspects of parental smoking during childhood were taken into account only by father’s smoking status when the children were 14 years old. Father’s smoking habits in pre- and postnatal periods were not known. Not being able to obtain all exposure information during or after pregnancy might raise the potential for residual confounding. Also, intensity of smoking during pregnancy was asked with a categorized question and could not be studied as a continuous variable. In addition, substance use at age 14 was a rare phenomenon in Northern Finland around the year 1980 which limited the power of the analysis. Furthermore, although the cohort was designed to study pregnancy risk factors relative to later life outcomes, it is worth noting that the present study utilized maternal smoking data from the 1960 s. Therefore, even though this ensured a long follow-up period, the results may not be fully generalizable to current time. Finally, information on smoking outcomes later in life was missing for high number of participants and analyses were conducted on imputed data. A previous study from the same birth cohort data has shown that high alcohol consumption, low educational level, unemployment, and being single at age 31 predicted lower participation at follow-up examination and questionnaires (Vladimirov et al., 2016). This can explain the small differences between main analyses and sensitivity analyses of complete case outcomes.

The strengths of this study were numerous. The dataset was prospective and based on a geographically representative, relatively large sample of 11,653 individuals. The follow-up was long, reaching from the mid-pregnancy until the fifth decade of life, and with multiple imputation we were able to minimize the possibility of selection due to missing data. Importantly, data were collected prospectively from the mothers during pregnancy, and from the offspring over their life course. Maternal smoking during pregnancy was reported in sufficient accuracy to perform a trajectory modelling based on monthly smoking data. Offspring’s smoking behavior could be assessed at several time points over their life course, and we could also address alcohol and substance use with our dataset. As the data collections comprised a wide range of lifestyle and health variables, we were able to account for several confounders in our multivariable analysis.

In this study we found four trajectories of maternal smoking during pregnancy which is in line with previous studies (Eiden et al., 2013, El-Khoury et al., 2017). Each trajectory represented a distinct phenomenon of maternal smoking during pregnancy, however, all trajectories represented either continuous behavior or smoking cessation. Among this population we did not identify any trajectories in which smoking was initiated or increased during pregnancy. However, the lack of such a trajectory is in line with previous studies (Eiden et al., 2013, El-Khoury et al., 2017, Dukes et al., 2017). Notably, the clear majority (i.e. 86%) of mothers did not smoke during the pregnancy. Even though the data were collected in 1960s, the prevalence of pregnancy smoking was similar to a recent *meta*-analysis (Berrueta et al., 2018). Mothers who smoked during pregnancy were more likely to be unmarried, to have a smoking partner, to be depressed, not to want the pregnancy, to be skilled workers, to live in urban areas, and to be younger than non-smoking mothers.

We found that all maternal smoking during pregnancy, even if quitted, associated with increased risk for offspring to smoke during their lives. One explanation to this finding might be that mothers who quit smoking during pregnancy may start it again after the childbirth and increase their children’s risk for smoking as role models. However, the current analysis did not associate maternal smoking with starting age or youth smoking, except for sensitivity analyses. In our sample, the starting age of smoking was around 16 years among the offspring of all maternal trajectories. Interestingly, maternal smoking trajectory associated with offspring’s smoked pack years by midlife. These findings suggest that offspring who have been exposed to continuous maternal smoking during pregnancy tend to smoke more heavily or for a longer period of time during their lifetime. A previous study has similarly found that maternal smoking does not associate with childhood smoking experimentation but associates with adult smoking (Paul et al., 2008). Another study has showed that maternal smoking during pregnancy associates with earlier onset of especially regular smoking in the offspring (D’Onofrio et al., 2012).

We found in this study that maternal smoking during pregnancy associates with offspring’s alcohol use at young age, which confirms the findings of previous studies (Griesler and Kandel, 1998, Hartman et al., 2006). However, in our study we could not adjust for parental alcohol use. Previous studies have showed that alcohol abuse and dependence transmit from parents to adolescent offspring (Li et al., 2002, Lieb et al., 2002, O'Brien and Hill, 2014) which may partly explain the association. Maternal alcohol use during pregnancy increases the risk for offspring alcohol and other substance use (Lieb et al., 2003).

The parent–offspring transmission of substance use behavior probably results from interactions between a wide range of biological, social and environmental risk factors. It is known that maternal smoking during pregnancy has harmful consequences on offspring’s substance use behavior and addiction (Brennan et al., 2002, Hellström-Lindahl and Nordberg, 2002, Al Mamun et al., 2006, Mumford et al., 2014) and previous studies have found evidence for a direct effect of maternal smoking during pregnancy on the development of offspring’s substance use (Melchior et al., 2010). However, mothers who smoke during pregnancy are more likely to smoke also during the childhood of the offspring (Scheffers-van Schayck et al., 2019), and maternal long-term smoking increases the risk of offspring’s smoking in youth (Riaz et al., 2018). Previous studies have shown that maternal smoking during pregnancy is strongly associated with the smoking habit of her partner, and sociodemographic, social, and psychological factors related to the family (O'Callaghan et al., 2006, Moor et al., 2015). These factors associate also with substance use behavior of the offspring (Macleod et al., 2008). In this current study, we found associations between maternal smoking trajectories and offspring’s alcohol use and smoking behavior even when father’s smoking and sociodemographic factors such as family’s occupational status, place of residence, marital status, mood of mother, and desirability of pregnancy were taken into account.

In conclusion, the present findings underline that to prevent parent-offspring transmission of smoking, the effective preventive measures and cessation support should start already when women are planning pregnancy. Also, our findings suggest that the negative effects of maternal continuous smoking during pregnancy might reach at least up to offspring’s middle age and include all substance use.

## Ethical considerations

The data were anonymized by the NFBC1966 data experts prior to analysis. Informed consent was collected at each stage from the participants and/or their legal guardians. The Declaration of Helsinki was followed, and approval was obtained from the Ethics Committee of the Northern Ostrobothnia Hospital District (94/2011). NFBC data is available from the University of Oulu, Infrastructure for Population Studies. Permission to use the data can be applied for research purposes via electronic material request portal. In the use of data, we follow the EU general data protection regulation (679/2016) and Finnish Data Protection Act. The use of personal data is based on cohort participant’s written informed consent at his/her latest follow-up study, which may cause limitations to its use. Please, contact NFBC project center (NFBCprojectcenter@oulu.fi) and visit the cohort website (www.oulu.fi/nfbc) for more information. The first author affirms that the manuscript is an honest, accurate, and transparent account of the study; that no important aspects of the study have been omitted; and that any discrepancies from the study as originally planned have been explained.

## CRediT authorship contribution statement

**Ina Rissanen:** Conceptualization, Methodology, Formal analysis, writing - original draft. **Markus Paananen:** Conceptualization, Methodology, Writing - review & editing, Supervision. **Terttu Harju:** Conceptualization, Writing - review & editing. **Jouko Miettunen:** Methodology, Investigation, Writing - review & editing, Project administration. **Petteri Oura:** Conceptualization, Methodology, Writing - review & editing, Visualization, Supervision.

## Declaration of Competing Interest

The authors declare that they have no known competing financial interests or personal relationships that could have appeared to influence the work reported in this paper.
